# Texting Older Sisters to Step (TOSS) Using Fitbits to Promote Physical Activity: A Feasibility Study

**DOI:** 10.1093/geroni/igab046.3261

**Published:** 2021-12-17

**Authors:** Pamela Bowen, William Opoku-Agyeman, Veronica Mixon, Olivia Affuso, Olivio Clay

**Affiliations:** 1 The University of Alabama at Birmingham, Birmingham, Alabama, United States; 2 Healthcare Management, University of North Carolina at Wilmington, North Carolina, United States; 3 UAB, Birmingham, Alabama, United States; 4 University of Alabama at Birmingham, Birmingham, Alabama, United States

## Abstract

Black women are disproportionately diagnosed with obesity (BMI ≥ 30 kg/m2). Obesity is a preventable but complex, public health problem that is multifaceted, chronic, and approximately 58% of Black women 60 years and older are classified as obese, compared to 38% of their White counterparts. This 12 week, pre/post, 2-group study aimed to determine if a peer-informed physical activity (PA) intervention with peer support would be feasible among community- dwelling, obese, older Black women to promote regular PA. Forty-eight potential participants were screened, 24 categorized as obese were enrolled and completed the study. The mean age was 64 (SD 3.0) years. Steps were measured by a Fitbit-Inspire with data successfully collected on 98% of days with the treatment group averaging a daily increase of 700-steps more than the control. Evaluation of intervention’s acceptability revealed that 100% enjoyed the study and using the Fitbit device. Text message readability was 100% and 95% said the study was motivational. Additionally, 8.3% said daily prompts were too frequent, 12% indicated that future studies should include additional social support, and 88% did not comment on the Fitbit community option for support, suggesting that this feature was not practical. Findings demonstrated that this intervention meets the criteria of being scalable, low cost, feasible, and acceptable for the older Black women. Using self-monitoring techniques in combination with at least one other behavioral strategy, such as our TOSS messages (cues for motivation) as the delivery channel for health promotion messages are a promising approach to increase PA behaviors.

